# Cognition after malignant media infarction and decompressive hemicraniectomy - a retrospective observational study

**DOI:** 10.1186/1471-2377-11-77

**Published:** 2011-06-23

**Authors:** Holger Schmidt, Trutz Heinemann, Judith Elster, Marija Djukic, Stefan Harscher, Katja Neubieser, Hilmar Prange, Andreas Kastrup, Veit Rohde

**Affiliations:** 1 University of Göttingen, Department of Neurology, Robert-Koch-Str. 40, D-37075 Göttingen, Germany; 2 University of Göttingen, Department of Medical Psychology & Medical Sociology, Waldweg 37A, D-37075 Göttingen, Germany; 3 Lichtenau Clinic, Department of Orthopedics, Am Mühlenberg, D-37235 Hessisch Lichtenau, Germany; 4 Evangelisches Krankenhaus Göttingen Weende, Department of Geriatrics, An der Lutter 24, D-37075 Göttingen, Germany; 5 Sophien- und Hufelandklinik, Department of Neurology, Henry-van-de-Velde-Straße 2, D-99425 Weimar, Germany; 6 University of Jena, Department of Neurology, Erlanger Allee 101, D-07747 Jena, Germany; 7 Klinikum Bremen-Mitte, Department of Neurology, St.-Jürgen-Str. 1, D-28177 Bremen, Germany; 8 University of Göttingen, Department of Neurosurgery, Robert-Koch-Str. 40, D-37075 Göttingen, Germany

## Abstract

**Background:**

Decompressive hemicraniectomy is a life-saving procedure for patients with malignant middle cerebral artery infarctions. However, the neuropsychological sequelae in such patients have up to now received little attention. In this study we not only describe neuropsychological deficits but also the quality of life and the extent of depression and other psychiatric symptoms in patients after complete media infarction of the non-speech dominant hemisphere.

**Methods:**

20 patients from two different university hospitals (mean ± standard deviation: 52 ± 14 years of age) who had undergone hemicraniectomy with duraplasty above the non-speech dominant hemisphere at least one year previously were examined using a thorough neurological and neuropsychological work-up. The quality of life and the extent of psychiatric problems were determined on the basis of self-estimation questionnaires. The patients were asked whether they would again opt for the surgical treatment when considering their own outcome. 20 healthy persons matched for age, gender and education served as a control group.

**Results:**

All patients but one were neurologically handicapped, half of them severely. Age was significantly correlated with poorer values on the Rankin scale and Barthel index. All cognitive domain z values were significantly lower than in the control group. Upon re-examination, 18 of 20 patients were found to be cognitively impaired to a degree that fulfilled the formal DSM IV criteria for dementia.

**Conclusions:**

Patients with non-speech dominant hemispheric infarctions and decompressive hemicraniectomy are at high risk of depression and severe cognitive impairment.

## Background

For more than a century, trephining of the skull to relieve intracranial pressure due to brain swelling has been a treatment option for space-occupying lesions such as large brain infarctions in the area supplied by the middle cerebral artery (MCA) [1]. With modern intensive care, the mortality rate dropped to 70% [2], and decompressive hemicraniectomy (DCH) has reversed the ratio of survival and death as compared to a conservative treatment regime alone. Further studies showed improved survival rate with DCH and better functional outcome [3-5]. Other authors have investigated the quality of life (QoL) after extensive MCA infarction [6,7] but to our knowledge only Leonhardt et al. [8] sufficiently described neuropsychological problems in patients after DCH (n = 14). The majority of their patients showed unexpectedly good results in some cognitive areas. However, they examined memory impairment only by one single subtest for non-verbal memory. Memory functions are crucial for social functioning, which is why memory impairment is an essential indicator of dementia. The primary aim of this study was to examine cognitive functions after DCH. We also described neurocognitive functions in DCH patients and correlated them with the assessment of QoL and the severity of mental symptoms.

## Methods

### Patients

Between 2004 and 2008, we contacted patients who had suffered from malignant infarction of the MCA and had been treated using DCH with duraplasty at a university medical center (Göttingen, Germany) between 1999 and 2003. A total of 58 patients were admitted to the Göttingen university clinic for malignant MCA infarctions during this time. In 1999, there were 18 patients, all of whom had been treated conservatively. Between 2000 and 2003, 40 patients with malignant MCA infarction were admitted, 19 of whom had been trephined and 21 treated conservatively. From 2000 on, patients with malignant MCA infarctions were also treated using DCH. All operated patients had infarctions of the non-speech dominant hemisphere. Seventeen of the operated patients could be re-examined; only one patient could not be located. Of the 39 conservatively treated patients, 18 died while still hospitalized. Seven of the conservatively treated patients could not be found for re-examination. In addition, we examined the 11 surviving patients with non-speech dominant hemispheric infarctions of the Jena University Hospital who comprised the patient population reported by S. Harscher [3] and who were operated on between 1996 and 2002. Three of these patients agreed to neuropsychological re-examination.

A prerequisite for inclusion in the study was that DCH had been performed at least one year before re-examination to allow for sufficient rehabilitation. All patients had infarctions of the non-speech dominant hemisphere (one was left-handed with a left-sided MCA infarction and intact speech ability). A control group of persons matched for age, gender and level of education was used since no normal values have been established for the extensive sequence of neuropsychological tests. The only normal values available for the tests used here are provided by handbooks for each single test. Exclusion criteria for the control group were the presence of neurological or psychiatric diseases, including substance abuse. The study was approved by the ethics committee of the University Medical Center Göttingen (approval no. 21102). The study was carried out in compliance with the Helsinki Declaration. All patients or their legal representatives gave informed consent before taking part in the study.

### Functional outcome

Patients were grouped according to the rating of their activities of daily life (ADL) using measurements established in stroke research [9-11], i.e. the Barthel index (BI) [12], and the Nottingham extended activities of daily living scale (NEADL) [13,14] (e.g.). Whether a cognitive disturbance meets the criteria for dementia is also dependent on the presence of impairment of the individual's social life and activities of daily living. Therefore, we used a subscore composed of the five items from the NEADL score ("NEADL_red._"), all of which we considered to be the most independent of concomitant physical handicap ("write letters", "make phone calls", "read books/newspapers", "money management", "social contacts"). Clinical outcome was assessed using the NIH stroke scale [15] (NIHSS).

### Neuropsychological assessment

Alertness was examined using the computerized TAP procedure [16], visual constructive functions were determined by a Rey-figure [17], verbal learning and memory were tested using the verbal learning test (VLT) [18], Wechsler memory scale (WMS-R) logical memory [19], WMS-R verbal pairs [19], and the German translation of the California verbal learning test (CVLT) [20]. Assessment of working memory was performed with the WMS III digit span test and spatial span test [21]. Impairments of non-verbal learning and memory were evaluated by the non-verbal learning test (NVLT) [22], WMS-R visual pairs [21], and the City map test [23]. We examined the frontal executive functions with the "Regensburger verbal fluency test with semantic alterations" [24], the Wisconsin card sorting test [25], Ruff's test [26] and the test for similarities (part of Hamburg-Wechsler Intelligence test for adults (HAWIE-R) [27]). The "Aachener Aphasie Test" (AAT) token test [28] was chosen to examine language impairment. All tasks in each test were presented and performed in the person's healthy field of vision, to rule out any error due to hemianopsia. Because of the extent of the test battery and the awareness that the handicapped patients might have problems with their attention functions, we placed the examinations of the domain attention in the early phase of the test sequence.

### Health related quality of Life (hrQoL)

The German version of the WHOQoL-Bref [29], a self-rated questionnaire, was used to measure the quality of life. In addition, the quality of sleep was measured using the Pittsburgh quality of sleep questionnaire (PSQI) [30].

### Grading of cognitive impairment

To evaluate the result of an entire neuropsychological domain we determined whether subtests were impaired, i.e. whether the z value was below -1.0 and thus below the first standard deviation under the mean of the normative data [31]. In addition, domains consisting of just one subtest (in this test battery, the domains of language and visuo-constructive functions) were considered to be impaired if the z values were lower than -2.0 [32]. There are various definitions for post-stroke dementia. In our study we chose the DSM IV criteria [33]. Together with social factors and daily life activities (which we quantified using the NEADL_red._), the DSM IV criteria require memory impairment and at least one further impaired domain.

In accordance with the literature, impaired memory was diagnosed if the patient's CVLT long delay, free recall test yielded a z value of less than -1 [34]. None of the patients had suffered from dementia or socially relevant cognitive decline before the hemispheric stroke occurred.

### Psychological symptom load

The reduced symptom checklist 90 [35] (SCL90r) was applied as a self-rating questionnaire for the evaluation of the psychological symptom load, comprising the subscales "somatization", "obsessive compulsive", "interpersonal sensitivity", " depression", "anxiety", "hostility", "phobic anxiety", "paranoid ideation", and "psychoticism". The evaluation yielded the global index measures "global severity index (GSI)", designed to measure overall psychological distress, the "positive symptom distress index (PSDI)", which measures the intensity of symptoms, and the "positive symptom total (PST)" that gives the number of self-reported symptoms. The degree of depression was quantified in addition to SCL90r scale 4 (depression) with the Beck's depression inventory (BDI) [36].

### Statistics

Data are given as mean and standard deviation, and cognitive data also include a 5-95% confidence interval (table 1). The Whitney-Mann U rank sum test was applied for group comparisons whenever Gaussian distribution was not present; otherwise, a t-test for independent samples was used. Spearman's r was calculated to analyze correlations. Frequencies were compared using Fishers's exact test in four-field tables unless more than four fields were present; in this case, the Chi^2^-test was calculated.

**Table 1:** Clinical and demographic data

*Pt.#*	*Sex*	*Handed-ness*	*Age at admission [years]*	*Affected territory*	*EarlyDCM*	*Stroke to follow-up [years]*	*Housing situation*	*Returned to work?*	Ambu-lation*	*Epi-lepsy*	*BI*	*mod. RS*	*NIHSS*	*NEADL*	*NEADL red.°*	*Repeat DCM?*
1	f	right	51,2	MCA, PCA	yes	1.5	at home	no	1	no	53	4	34	42	16	yes

2	m	right	49,7	MCA	yes	2.2	nursing home	no	1	no	42	4	14	38	15	no

3	m	both	61,3	MCA	no	2.4	at home	no	1	no	74	3	2	55	17	yes

4	m	right	38,8	MCA	no	2.1	at home	no	1	yes	83	3	10	16	13	yes

5	m	right	68,0	MCA, ACA	no	3.0	at home	n.a.	2	no	24	4	9	31	8	yes

6	f	right	66,9	MCA	yes	1.7	at home	n.a.	4	no	10	4	15	26	7	no

7	m	right	35,7	MCA, ACA	no	4.1	at home	no	1	no	76	3	8	31	16	yes

8	m	right	69,7	MCA, ACA	no	0.8	at home	n.a.	4	no	6	5	18	23	5	yes

9	m	right	66,9	MCA	yes	1.5	at home	n.a.	4	yes	22	4	10	34	14	yes

10	m	right	46,7	MCA	yes	1.7	at home	no	0	yes	80	4	9	56	18	yes

11	f	right	13,8	MCA, ACA	no	2.9	at home	yes	0	no	96	3	6	77	19	yes

12	m	left	45,9	MCA	yes	2.5	nursing home	no	3	yes	77	4	13	30	10	no

13	f	right	49,5	MCA	no	2.9	at home	no	1	no	95	0	5	42	16	yes

14	m	right	45,1	MCA	yes	2.9	at home	no	1	yes	51	4	11	37	13	yes

15	m	right	46,0	MCA	no	6.1	at home	no	1	no	40	4	15	85	20	no

16	m	right	51,8	MCA	no	7.9	nursing home	no	4	no	10	5	7	33	10	no

17	m	right	60,8	MCA	yes	4.7	at home	no	1	yes	39	4	10	39	15	yes

18	m	both	45,5	MCA	yes	5.0	at home	no	1	yes	3	4	14	28	7	yes

19	m	right	74,0	MCA	yes	1.5	at home	n.a.	3	yes	13	4	9	32	6	no

20	m	right	63,7	MCA	yes	1.9	at home	no	3	yes	60	4	9	46	15	no

* Coding for ambulation: 0 = normal or slightly impaired, 1 = significantly impaired, 2 = independent only for a short distance, 3 = manages short distance only with help, 4 = immobile

NEADL subscale with items that are independent from physical handicap (maximum 20 points)

## Results

### Patients and control group

The patient group (P) and the group of 20 healthy control persons (C) were matched for age (mean ± SD 55.5 ± 13.7_P _vs. 53.5 ± 13.0 years_C_; p = 0.65), sex (m/w 16/4_P _vs. 15/5_C_, p = 1) and education (9.9 ± 1.6_P _vs. 10.4 ± 2.3_C _school years, p = 0.4). The age at neuropsychological examination was 55.4_P _± 13.7 and 53.5_C _± 13.0 years, respectively (p = 0.65). In the group of patients the mean interval from the first symptoms to DCH was 45.8 ± 60 hours (median [25^th^/75^th^] 21.8 [15.6/52.5]). Eleven of the patients had undergone DCH in the early course (< 24 h after onset of symptoms) and in 9 patients, DCH had been delayed.

Re-examination of patients was carried out at a mean ± SD 3.0 ± 0.8 (median [25^th^/75^th^] 2.4 [1.7/3.5]) years later.

In agreement with the literature, the patients in this collective with malignant MCA infarction survived significantly more often with neurosurgical than with conservative treatment (18 of 19 vs. 21 of 39 hospital survivors, p = 0.0014). Upon re-examination of the patients who received DCH, 17 patients lived at home; three were living in a nursing home. Nine of 20 patients had developed symptomatic epilepsy, and 13 had been diagnosed with post-stroke depression; 11 of them were treated at the time of examination with anti-depressive agents. Confronted with the hypothetical situation of turning back time to the decision to undergo DCH, the majority of patients stated that they would again opt for a decompressive hemicraniectomy (13 "yes" vs. 7 "no"; Table 1). This statement was independent of the presence of cognitive impairment (Fisher's exact test p = 0.58).

### Neurological outcome

At the time of re-examination the median [25^th^/75^th ^percentile] of the NIHSS was 10 [8.5/14] and 11 patients had scale values of ≥ 10. Ten of 19 patients showed a Barthel index greater than 50 (one patient with bilateral leg amputation before the stroke occurred was excluded), and the median [25^th^/75^th ^percentile] was 46.5 [17.5/76.5]. The median modified Rankin scale was 4 (3.5/4.0; 25^th^/75^th ^percentile), and 5 patients had a mRS < 4 while 15 patients were affected severely (mRS 4 and 5).

The majority (16 patients) was mobile in their respective home settings, but only two displayed normal ambulation.

### Neuropsychological outcome

**Figure 1:**
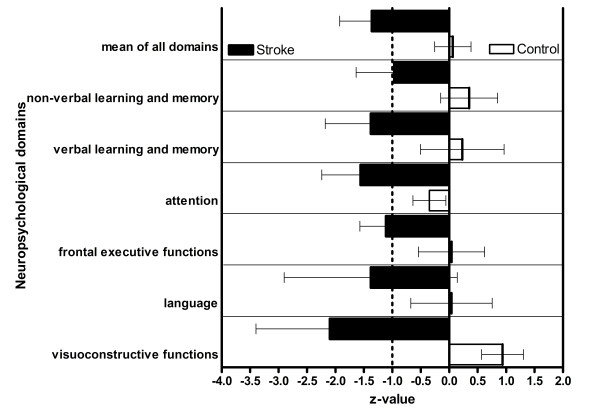
**Neuropsychological domain mean ± SD z values (all domains of the patients group are significantly different from the control group)**.

Four of 20 patients left out some tasks of the test battery, more than in the healthy control group (0/20). The results of all subtests are summarized in Table 2. For all subtests, the results of the patient group were significantly worse than those of the control persons (Table 1). The mean ± SD z values of the respective domains are given in Figure 1. No subject of the control group fulfilled the criteria of abnormal cognitive functions. The differences in cognitive functioning between patients with and those without depression or epilepsy were not significant. Eighteen of 20 patients fulfilled the formal criteria for dementia according to the DSM IV; one of them demonstrated abnormal cognitive performance but only a slightly reduced NEADL and a normal NEADL_red. _score (and thus may be considered as a borderline case with only minimal cognitive impairment).

**Table 2:** Neuropsychological test results

	*Stroke group*			*Control group*			
***z-values***	***valid N***	***mean ± SD***	***CI [5% - 95%]***	***valid N***	***mean ± SD***	***CI [5% - 95%]***	***p-value***

CVLT trial 1	20	-1.1 ± 1.2	[1.6 - -0.5]	20	-0.2 ± 1.0	[-0.6 - 0.3]	0.0056

CVLT trial 5	20	-2.2 ± 1.1	[-2.7 - -1.7]	20	0.2 ± 1.4	[-0.4 - 0.8]	< 0.0001

CVLT sum 1-5	19	-2.0 ± 1.1	[-2.5 - -1.5]	20	0.1 ± 0.3	[-0.1 - 0.2]	0.0001

CVLT list B	20	-1.6 ± 1.2	[-2.1 - -1.0]	20	-0.5 ± 1.4	[-1.2 - 0.2]	0.0144

CVLT short delay free recall	20	-1.8 ± 1.1	[-2.3 - -1.2]	20	0.0 ± 1.0	[-0.4 - 0.5]	0.0001

CVLT short delay cued recall	20	-1.6 ± 1.3	[-2.1 - -1.0]	20	0.0 ± 1.2	[-0.5 - 0.5]	0.0007

CVLT long delay free recall	20	-1.5 ± 1.3	[-2.1 - -0.9]	20	0.3 ± 1.2	[-0.2 - 0.8]	0.0003

CVLT long delay cued recall	20	-1.5 ± 1.2	[-2.1 - -0.9]	20	0.2 ± 1.0	[-0.2 - 0.6]	0.0001

CVLT semantic cluster ratio	19	-1.4 ± 1.2	[-2.0 - -0.9]	20	-0.3 ± 1.3	[-0.9 - 0.3]	0.0070

CVLT serial cluster ratio	20	1.9 ± 1.4	[1.2 - 2.6]	20	0.5 ± 1.2	[-0.1 - 1.1]	0.0047

CVLT slope	20	-1.1 ± 1.0	[-1.6 - -0.6]	20	0.2 ± 1.1	[-0.3 - 0.7]	0.0014

CVLT perseverations	20	0.6 ± 0.9	[0.1 - 1.0]	20	0.6 ± 0.8	[0.2 - 0.9]	0.8181

CVLT recognition hits	20	-1.1 ± 1.1	[-1.6 - -0.5]	20	0.0 ± 1.1	[-0.6 - 0.5]	0.0077

CVLT false positive	20	-1.0 ± 1.3	[-1.6 - -0.4]	20	0.1 ± 0.3	[0.0 - 0.2]	0.0071

TAP alertness w/o audio alert; median reaction time	18	-2.2 ± 0.9	[-2.6 - -1.7]	20	-0.2 ± 0.8	[-0.6 - 0.1]	< 0.0001

TAP alertness w/o audio alert; reaction time SD	18	-1.7 ± 1.3	[-2.3 - -1.1]	20	0.5 ± 1.5	[-0.2 - 1.2]	0.0001

TAP alertness with audio alert; median reaction time	18	-2.2 ± 1.0	[-2.7 - -1.7]	20	-0.3 ± 0.8	[-0.7 - 0.1]	< 0.0001

TAP alertness with audio alert; SD reaction time	18	-1.5 ± 1.3	[-2.1 - -0.8]	20	0.2 ± 0.7	[-0.1 - 0.6]	0.0002

TAP divided attention median reaction time	18	-2.0 ± 1.1	[-2.5 - -1.5]	20	-1.1 ± 0.7	[-1.5 - -0.8]	0.0008

TAP divided attention SD reaction time	18	-1.8 ± 1.0	[-2.3 - -1.3]	20	-0.2 ± 0.9	[-0.7 - 0.2]	0.0001

TAP divided attention omissions	18	-2.7 ± 0.9	[-3.2 - -2.3]	20	-0.2 ± 0.6	[-0.5 - 0.1]	< 0.0001

TAP go-no-go median reaction time	18	-1.4 ± 1.9	[-2.4 - -0.5]	20	-0.5 ± 0.6	[-0.8 - -0.2]	0.0052

TAP go-no-go reaction time SD	18	0.0 ± 1.3	[-0.7 - 0.6]	20	2.5 ± 0.8	[2.1 - 2.9]	< 0.0001

TAP go-no-go errors	18	-0.3 ± 1.1	[-0.9 - 0.2]	20	0.1 ± 0.7	[-0.2 - 0.5]	0.2482

WCST concepts	20	-1.2 ± 1.1	[-1.7 - -0.7]	20	0.0 ± 1.0	[-0.5 - 0.5]	0.0013

WCST reaction time	20	1.8 ± 3.2	[0.3 - 3.2]	20	0.0 ± 1.0	[-0.5 - 0.5]	0.0133

WCST errors	20	-0.8 ± 1.3	[-1.3 - -0.2]	20	0.0 ± 1.0	[-0.5 - 0.5]	0.0639

WCST perseverations	20	0.9 ± 1.9	[0.0 - 1.7]	20	0.0 ± 1.0	[-0.5 - 0.5]	0.0167

CFT copy	19	-2.2 ± 1.2	[-2.8 - -1.6]	20	0.9 ± 0.4	[0.8 - 1.1]	< 0.0001

CFT recall 30 min	19	-1.2 ± 0.9	[-1.7 - -0.8]	20	0.8 ± 0.6	[0.5 - 1.1]	< 0.0001

CFT %recall	18	-0.4 ± 1.5	[-1.1 - 0.4]	20	0.6 ± 0.6	[0.4 - 0.9]	0.0040

CFT copy-recall	19	1.0 ± 1.4	[0.3 - 1.6]	19	0.5 ± 0.7	[0.2 - 0.8]	0.0726

NVLTR true positive	20	-1.4 ± 1.7	[-2.1 - -0.6]	19	-0.2 ± 1.1	[-0.7 - 0.4]	0.0076

NVLT false positive	20	-0.6 ± 1.7	[-1.4 - 0.2]	19	-0.7 ± 0.7	[-1.0 - -0.3]	0.4397

Difference t+/f+ NVLT	20	-2.1 ± 0.9	[-2.6 - -1.7]	19	-0.7 ± 0.9	[-1.1 - -0.3]	0.0001

VLT true positive	20	-1.6 ± 1.4	[-2.3 - -1.0]	19	0.2 ± 1.2	[-0.3 - 0.8]	0.0006

VLT false positive	20	-0.2 ± 1.5	[-0.9 - 0.5]	19	-0.2 ± 0.9	[-0.6 - 0.2]	0.7466

Difference t+/f+ VLT	20	-1.5 ± 1.4	[-2.1 - -0.9]	19	-0.1 ± 0.8	[-0.5 - 0.2]	0.0033

LGT city map test	20	-2.1 ± 0.8	[-2.5 - -1.8]	19	0.0 ± 1.0	[-0.5 - 0.5]	< 0.0001

WMS logical memory I	20	-1.5 ± 1.5	[-2.2 - -0.8]	20	0.7 ± 1.0	[0.2 - 1.1]	0.0001

WMS logical memory II	20	-1.8 ± 1.2	[-2.3 - -1.2]	20	0.6 ± 0.8	[0.2 - 1.0]	< 0.0001

WMS visual pairs I	19	-0.8 ± 1.4	[-1.5 - -0.1]	19	0.4 ± 1.1	[-0.1 - 0.9]	0.0094

WMS visual pairs II	19	-0.8 ± 1.5	[-1.6 - -0.1]	19	0.7 ± 0.5	[0.5 - 1.0]	0.0017

WMS verbal pairs I	19	0.4 ± 1.5	[-0.4 - 1.1]	20	0.7 ± 1.1	[0.2 - 1.2]	0.5741

WMS verbal pairs II	18	-0.9 ± 1.5	[-1.6 - -0.1]	20	0.3 ± 0.9	[-0.2 - 0.7]	0.0179

HAWIE similarities	17	-0.5 ± 1.1	[-1.1 - 0.1]	20	1.5 ± 1.3	[0.9 - 2.0]	0.0001

5-point test	17	-2.6 ± 0.7	[-2.9 - -2.2]	20	-0.2 ± 1.1	[-0.7 - 0.3]	< 0.0001

WMS digit span (forward)	17	-0.1 ± 1.4	[-0.8 - 0.6]	20	0.6 ± 0.9	[0.2 - 1.1]	0.0722

WMS digit span (backwards)	17	-1.1 ± 1.4	[-1.8 - -0.3]	20	-0.2 ± 1.2	[-0.8 - 0.3]	0.0148

Corsi block span (forward)	17	-1.9 ± 1.0	[-2.4 - -1.4]	20	0.0 ± 1.2	[-0.5 - 0.6]	0.0001

Corsi visual span (backwards)	17	-2.5 ± 0.6	[-2.9 - -2.2]	20	-0.4 ± 0.9	[-0.8 - 0.0]	< 0.0001

RWF verbal fluency	20	-1.3 ± 0.8	[-1.7 - -1.0]	20	0.3 ± 0.7	[0.0 - 0.6]	< 0.0001

RWF semantic verbal fluency with alternations	20	-1.5 ± 0.8	[-1.9 - -1.2]	20	0.5 ± 0.6	[0.2 - 0.8]	< 0.0001

AAT mistakes	19	-1.2 ± 1.8	[-2.0 - -0.3]	20	0.1 ± 0.7	[-0.3 - 0.4]	0.0264

Descriptive data for patient group and control group, comparison performed with the Whitney-Mann U-test

Even with a very conservative definition of neuropsychological impairment (z < -1.5), 16 of 20 patients still met the definition of dementia in accordance with DSM IV.

### Influence of age

Age correlated significantly negative with the BI (r = -0.58; p = 0.007) and with a higher mRS (0.49; p = 0.03). Visuo-constructive functions (r = -0.51; p = 0.02) and the physical quality of life in the WHOQoL-Bref questionnaire (r = -0.59; p = 0.006) were correlated negatively with the patients' age at admission, whereas the subjective physical quality of life was not correlated with age (r = 0.05; p = 0.8). There was also no correlation between the age at admission and the time interval between stroke onset and surgery.

### Glasgow Coma Scale (GCS) upon admission

Lower GCS at the time of admission was significantly correlated with higher scores in the PSQI, i.e. with poorer sleep quality after convalescence. We could not find any further associations.

### School years

The years of basic education were positively correlated for subscales indicative of verbal learning (CVLT trial 5, sum 1-5, slope), with subtests associated with frontal executive functions (CVLT cluster, TAP go-no-go) and also with the z value of domain values for attention and verbal memory.

### Psychological sequelae

The mental symptom load for all domains analyzed by SCL90r was higher in patients compared with healthy controls. These differences reached statistical significance for the domains "somatization", "obsessive-compulsive", "depression", "anxiety", "phobic anxiety", and "psychoticism". However, only for the subscale "depression" were these differences higher than one standard deviation. As compared to the control group, depressive mood represents the main mental problem in patients after DCH (median [25^th^/75^th ^perc.] z value -3 [-3/-1.2]_P _vs. -0.02 [-0.4/0.77]_C_, p < 0.001). Thirteen of 20 patients after DCH were depressive (compared to none in the control group). Of our patients, only half were treated with anti-depressive drugs. No patient underwent psychotherapy. BDI z values from patients without epilepsy were not significantly lower than those of patients with epilepsy (-3 [-3/-2.77]_P_-1.98 [-3/1.16]_C_, p = 0.23).

### Health-related Quality of Life

**Figure 2:**
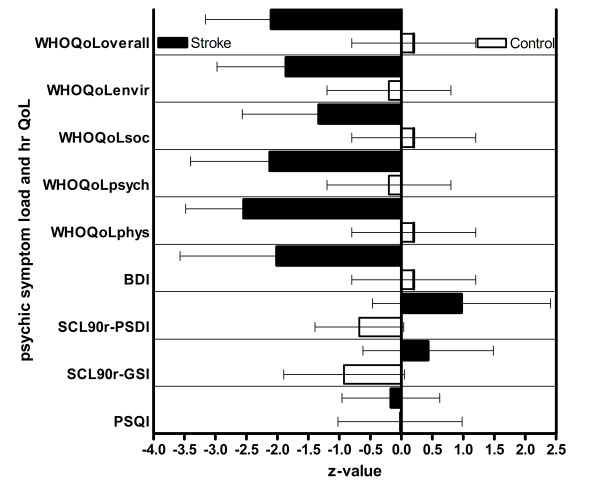
**Domain mean ± SD z values of quality of life, sleep quality and mental symptom load (all domains of the patients group are significantly different from the control group)**.

In all domains the analysis of the WHOQoL Bref questionnaire showed results indicative of lower quality of life in patients compared with the control group (Figure 2). Mean z values for the WHOQoL questionnaire displayed a clinically significant impairment of the health-related QoL. The WHOQoL-Bref subscales and the overall WHOQoL score were not significantly different in the stroke patient group with respect to the level of education (WHOQoL-Bref_overall_: Kruskal-Wallis p = 0.16) but they were correlated significantly with the BDI z values (r = 0.7, p < 0.001). All WHOQoL subscales and the WHOQoL-Bref_overall _score were positively correlated with each cognitive domain.

## Discussion

That DCH can be a life-saving treatment option for complete MCA stroke is no longer a matter of debate [3,5,9-11,37-39]. However, there are only limited neuropsychological data on patients who underwent decompressive hemicraniectomy after complete MCA infarction. Only one previous study of 14 patients who underwent DCH focused on neuropsychological sequelae [40]. In contrast to the promising data on survival for DCH, our findings on impairment of higher cortical functions after DCH were not at all encouraging: More than two-thirds of our patients met the criteria for dementia after the hemispheric stroke. In contrast to Leonhardt et al. [40] who found that mean visuo-spatial and visuo-constructive capabilities were nearly normal and self-assessed mood not at all impaired, none of the cognitive domain z values in our patient group fell within the normal range. The only results comparable to ours were for attention and non-verbal memory. The reason for the discrepancy between our findings and Leonhardt et al. is that they did not focus on memory and learning and - after observing the difficulties patients had with tests for attention and psychomotor speed -they just eliminated most of the tests for this domain.

The self-assessment of mood in our patient group revealed significant impairments. In contrast to Leonhardt [8], but congruent with other authors [9,41], we found increased scores for the BDI, although the majority of the patients had received anti-depressive drugs. These results can be clearly explained by previous findings on right-sided brain pathology: Right-sided hemispheric lesions impair both verbal and non-verbal memory and learning [42,43]. In patients who underwent right-sided surgical pallidotomy, one observed, in addition to learning deficits for verbal and non-verbal content, a deterioration of frontal executive and visuo-constructive functions [44]. Patients with right-sided fronto-temporal lobe atrophy have problems with spatial memory [45] and are also prone to develop affective disorders such as depression [46] or problems with the emotional processing [47-50]. The results in our patients after massive stroke-induced destruction of the right hemisphere underline the importance of this side of the brain for cognition and mood.

In regard to cognitive outcome after DCH, our results may be too negative since 8 of our patients were older than 60 years, whereas other recent studies included only patients up to 60 years [11]. Furthermore, the current guidelines of the European Stroke Organization (ESO) recommend a maximum cut-off age of 60 years for the DCH [51].

The higher age of our study population also explains the high proportion of patients with an unfavorable Rankin scale (75% in our study) compared to 43% in pooled data from the DESTINY, HAMLET and DECIMAL trials. Thus, the population analyzed here is certainly not comparable to the study groups in the above mentioned studies on DCH. The same is true for the variance of time from stroke onset to the neuropsychological tests. Therefore, our data should be interpreted cautiously with respect to these issues and might not be valid for a younger population examined within a narrower time interval after the treatment. Our findings are comparable to another study with a similar rate of unfavorable outcome (73.7%) [7] which also included older patients. Furthermore, our data on indicators of activities of daily living (NEADL, BI) may also not be representative of a younger population. Other studies which included older patients also found these indicators to be correlated negatively with age [7].

Another limitation of our investigative focus on cognitive deficits is that patients with speech-dominant hemispheric stroke were not included. Our findings for the depression questionnaires, therefore, are not transferable to patients with DCH over the speech-dominant hemisphere, as it is well known that mood disturbances appear more often in patients with right-sided stroke than in those with left-sided cerebral ischemia [43,47]. Strategically unfavorable localizations of small right-sided strokes, i.e. in the frontal projections of the corpus callosum, can cause major depression [49]. The same is true for cognitive functions; neuropsychological investigations of patients after neurosurgical interventional treatment of Parkinson's disease have shown the importance of the right brain for cognitive functions [44,52].

In regard to both mood and cognition, our findings may significantly differ from patients with left-sided MCA infarctions and DCH. Since this study focused on cognitive functioning, reliable results were dependent on the patient's understanding of the test rules. The degree of comprehension is extremely difficult to quantify in partially aphasic patients, and there is no general rule as to how to correct the cognitive results for the individual type of aphasia. Therefore, we decided to exclude patients with speech-dominant hemispheric infarctions rather than misinterpreting their cognitive test results.

As in other studies on DCH, most of our patients were able to walk after DCH and had regained some independence, despite the persisting physical handicaps [11]. Physical impairment is, however, just one element which affects human well-being; in our patient group, the quality of life was significantly lower than in healthy control patients, which matches the findings reported by other groups [4,53,54]. Not surprisingly, in our patients, hrQoL was significantly correlated with the Beck's depression inventory. This underlines the necessity to diagnose and to treat post-stroke depression early and effectively in such patients. Depression is often a cause of cognitive disturbances, and vice versa. Our data do not allow us to safely quantify the influence of depressive pseudo-dementia on the test results. However, since cognition and mood interact closely, it is very difficult or perhaps impossible to differentiate their mutual influences. The lack of a significant correlation between the BDI and cognitive functioning, however, indicates that depression was not the primary factor affecting cognitive performance in DCH patients. In addition to the lack of a significant correlation of cognitive scores with the BDI, our patients also showed abnormal results for verbal learning and visual constructive tests. These domains are usually spared in depressive pseudo-dementia [55,56].

The presence of symptomatic epilepsy should have an influence on cognitive functioning as well as mood [57]. Particularly right-sided brain pathology should predispose patients with epilepsy for depression [58]. It is most likely that the sample size of our study population was too small to reproduce these effects.

All told, the findings demonstrate that the probable outcome after DCH of the non-speech dominant hemispheric infarction includes significant cognitive deficits along with a physical handicap and impaired mood, and consequently, a diminished quality of life. The prospect of living with depression combined with severe physical and cognitive handicaps may be inacceptable for many patients; for others, however, it might be the better option compared to 70-80% mortality with a conservative treatment [59].

A significant limitation of this study in regard to the discussion of post-hoc patient agreement is the lack of information on long-term cognitive deficits in patients who survived a conservative treatment. It is possible that neurological and cognitive sequelae in these patients are higher than in those who had been operated on due to the untreated swelling of the ischemic brain regions. To our knowledge no such data are available. The group of the operated patients who stated that if they had to decide again, they would **not **have opted for DCH did not differ significantly in their neuropsychological test results, and also did not display lower quality of life scores as compared to the patients who stated that they would again opt for DCH. However, the former patient group suffered from significantly higher scores for depressive mood (p = 0.039) than the latter. More than half of our patients were treated with anti-depressive drugs. It could be hypothesized that the patients whose post-hoc assessment of DCH was negative just needed a better anti-depressive treatment, especially since the agreement rates in our patient group were considerably lower than in other trials (e.g. [5]). Our data can neither confirm nor reject this hypothesis. The fact that there was no difference in BDI score between the patients who were administered anti-depressive drugs and those who were not might argue against this idea. However, all reported rates of retrospective agreement with DCH could be severely biased by the interview situation. The patients were interviewed by a person who was involved in their stroke treatment and thus in the life-saving process, which may have inhibited them from answering honestly, at the risk of appearing ungrateful.

## Conclusion

The fact that in the majority of patients a significant cognitive decline is to be expected should not lead to a general rejection of DCH as a treatment option. The problem for the treating physician is to ascertain whether the patient is willing to risk death with conservative treatment or survive to a life of coping with impaired neurocognitive functions, probable depression and diminished quality of life. Ideally, this decision should be made by the patients themselves, which is too often not possible.

## Competing interests

The authors declare that they have neither competing financial nor non-financial interests with respect to this manuscript.

## Authors' contributions

HS conceived and coordinated the study and drafted the manuscript, TH carried out the neuropsychological examinations and took part in the study design, JE coordinated and carried out the statistical analysis, MD was involved in the study design and the neurological examinations, SH participated in the coordination of the study and drafted the manuscript, KN carried out the statistical analysis and helped coordinate the study, HP and AK participated in study design, and VR participated in the study design as well as coordination.

All authors have read and approved the final manuscript.

## Pre-publication history

The pre-publication history for this paper can be accessed here:

http://www.biomedcentral.com/1471-2377/11/77/prepub
